# Cutaneous Injury and *Vibrio vulnificus* Infection

**DOI:** 10.3201/eid1208.051495

**Published:** 2006-08

**Authors:** P.H. Chung, S.K. Chuang, Thomas Tsang, Lai Wai-man, Raymond Yung, Janice Lo, for the Collaborative Study Group on Vibrio vulnificus Infection in Hong Kong

**Affiliations:** * Department of Health, Hong Kong Special Administrative Region, People's Republic of China;; † Hospital Authority, Hong Kong Special Administrative Region, People's Republic of China;; ‡ Centre for Health Protection, Hong Kong Special Administrative Region, People's Republic of China

**Keywords:** *Vibrio vulnificus*, seafood, emerging, letter

To the Editor: Vibrio vulnificus infection is transmitted by eating contaminated seafood or by exposure to seawater through an open wound (*1*). Among immunocompromised persons, especially those with chronic liver disease, V. vulnificus can cause a life-threatening illness characterized by blistering skin lesions, necrotizing fasciitis, and septic shock (*2**–**5*). However, the epidemiology and risk factors for severe forms of V. vulnificus infection among healthy persons are less well documented (*4**–**6*).

We conducted a retrospective clinical record review of V. vulnificus infections in persons admitted to all public hospitals from January 1, 2003, through August 31, 2005, in Hong Kong, which has a population of >6 million persons. We defined a case-patient as a patient with culture of V. vulnificus from any clinical specimen. A record search of clinical case notes was performed through a computerized clinical management system maintained by the Hospital Authority, which manages all public hospitals in Hong Kong. For each case-patient identified, we reviewed demographic data (age, sex, occupation, residence), clinical and laboratory data (date of onset, symptoms, laboratory investigation findings, diagnosis, outcome), and potential risk factors (past health and possible source of exposure) associated with the case. We compared previously healthy patients with patients who had predisposing medical conditions in terms of demographic profile, clinical signs and symptoms and outcome, and known exposure factors. Mann-Whitney U tests, χ^2^ tests, or Fisher exact tests were used to detect significant differences (α = 0.05).

We identified 29 cases over the 32-month study period. Twenty-two (76%) patients had disease onset from May through August, the summer season in Hong Kong. Fifteen (52%) cases were in men, and the median age was 70 years (range 24–82 years). Fifteen (52%) patients had underlying illnesses that were known to predispose them to V. vulnificus infection, including chronic liver disease (30%), chronic renal failure (15%), diabetes mellitus (7%), and thalassemia major (3%). Fourteen (48%) patients were previously healthy. No significant differences in age and sex were found.

Among the 14 previously healthy patients, the consequences of V. vulnificus infection included necrotizing fasciitis (70%), severe cellulitis (7%), primary septicemia (14%), and gastroenteritis (7%). Two patients who had necrotizing fasciitis and 1 patient with primary septicemia died. Compared with patients with predisposing medical conditions, patients with a history of good health had a higher (but not significant) proportion of necrotizing fasciitis (70% vs 47%, p = 0.12), a lower proportion of septicemia (14% vs. 27%, p = 0.26), and an equal number of severe cases of cellulitis (7% vs. 7%). Furthermore, fewer patients with a history of good health died than did patients with predisposing illnesses (21% vs. 33%, p = 0.25). The median duration between symptom onset and admission for all patients was 1 day (range 0–3 days), with no significant difference between the 2 groups.

A history of cutaneous injury or a skin prick from a seafood part (e.g., fish fin, shrimp spine, or crab leg) was significantly more common among previously healthy patients than among patients with predisposing illnesses (70% vs. 27%, p = 0.02). Ten (83%) of the 12 previously healthy patients with necrotizing fasciitis and septicemia reported a history of cutaneous injury. The corresponding proportion was significantly lower (31%) among patients with predisposing medical conditions (p = 0.01). Among all 29 patients, a history of eating raw oysters or other raw or undercooked seafood before illness onset was uncommon and was only reported by 1 patient. Although V. vulnificus has not been proven as the cause of gastroenteritis, Hseuh et al. have suggested that such results might have occurred because patients with diarrhea seldom sought care from a large teaching hospital or saved stool samples for investigation (*7*).

V. vulnificus infection was first reported in humans in 1979 (*1*). Since then, most case reports have focused on immunocompromised persons and their risk from eating raw oysters among (*4**–**6*). Our study found that a considerable proportion of V. vulnificus infections in Hong Kong occur among healthy persons. Furthermore, severe forms of the infection, such as necrotizing fasciitis and septicemia, are relatively common among healthy persons, although they may cause fewer deaths than they do among persons with predisposing medical conditions. Among healthy persons, V. vulnificus infection is most likely associated with a history of cutaneous injury caused by handling seafood, which can allow the bacteria to enter the body through an open wound. The risk of exposure is more important in this locality than in other areas where swimming or eating raw oysters and undercooked seafood are the major risk factors (*4**,**6**–**8*), possibly because fresh seafood is widely consumed, and seafood is easily accessible in wet markets in Hong Kong. Our study shows that the risk is higher during the summer, which is consistent with the fact that V. vulnificus is more active in warmer temperatures (*9*). We suggest that all persons, even healthy persons, exercise caution to avoid injury while handling seafood. Physicians should consider possible V. vulnificus infection when diagnosing a rapidly progressive skin and soft tissue infection in a healthy person who reports an injury from handling seafood.
